# Label Fusion Strategy Selection

**DOI:** 10.1155/2012/431095

**Published:** 2012-02-06

**Authors:** Nicolas Robitaille, Simon Duchesne

**Affiliations:** ^1^Centre de Recherche de l'Institut Universitaire en Santé Mentale de Québec, 2601, Chemin de la Canardière, QC, Canada G1J 2G3; ^2^Radiology Department, Faculty of Medicine, Laval University, Pavillon Ferdinand-Vandry, 1050, Avenue de la médecine, QC, Canada GIV 0A6

## Abstract

Label fusion is used in medical image segmentation to combine several different labels of the same entity into a single discrete label, potentially more accurate, with respect to the exact, sought segmentation, than the best input element. Using simulated data, we compared three existing label fusion techniques—STAPLE, Voting, and Shape-Based Averaging (SBA)—and observed that none could be considered superior depending on the dissimilarity between the input elements. We thus developed an empirical, hybrid technique called SVS, which selects the most appropriate technique to apply based on this dissimilarity. We evaluated the label fusion strategies on two- and three-dimensional simulated data and showed that SVS is superior to any of the three existing methods examined. On real data, we used SVS to perform fusions of 10 segmentations of the hippocampus and amygdala in 78 subjects from the ICBM dataset. SVS selected SBA in almost all cases, which was the most appropriate method overall.

## 1. Introduction

Label fusion is a process used in medical image segmentation. Its aim is to produce a single, discrete element or *label* from a combination of multiple independent inputs. The merged result is potentially more accurate, with respect to the exact, sought segmentation, than each individual input due to the reduction of uncorrelated errors. Labels can be obtained by combining inputs from different raters or automated segmentations [1, 2].

A long-term goal of our research program is to obtain accurate, automated segmentations of neuroanatomical structures, primarily the hippocampus (HC). Our primary motivation stems from our work in Alzheimer's disease, for which HC volume and atrophy measurements are putative disease markers (see reviews in [3–6]). Of the multiple HC segmentation approaches available (see [7] for review), novel template-based paradigms propose the use of template libraries [8]. In such approaches, a single label is found by combining multiple individually segmented HC through label fusion [2]. 

To reach our goal, we thus decided to investigate different fusion processes. To suit our research context, we restricted our analysis to techniques that depend solely on given input labels. We disregarded techniques that depend on intensity images [9, 10], since these images may sometimes be unavailable or noisy. We also ignored techniques that depend on object-specific training, i.e. that have geometric or topological prior. 

Our first objective was to characterize applicable label fusion strategies. The first approach is the Vote method (or sum rule), which has been widely used and described by virtue of its simplicity [1, 9, 11–13]. The second is also a well-known technique called Simultaneous Truth and Performance Level Estimation (STAPLE), initially proposed by Warfield et al. [14, 15], and used in a variety of studies [9, 16]. The third approach is referred to as Shape-Based Averaging (SBA), which incorporates spatial information [17].

While testing the implementations of these three approaches on simulated data, we observed that the technique with a result closest to the ground truth was not the same depending on the dissimilarity between raters' input labels, as detailed below. Therefore, the second objective of our study was to propose an empirical, hybrid STAPLE-Vote-SBA (SVS) technique that automatically selects the right label fusion approach based on this dissimilarity.

We report results of comparison tests on the four label fusion methods for simulated two-dimensional (2D) and three-dimensional (3D) data as well as HC and amygdala (AG) labels obtained from magnetic resonance images (MRI). All images used in this study were binary. For the real data, we performed label fusion on HC and AG independently.

## 2. Materials and Methods

### 2.1. Mathematical Notation

Our mathematical notation is as follows. We consider an image of *N* pixels or voxels (*x* = 1,2,…, *N*) for which *K* raters (*k* = 1,2,…, *K*) each produces a binary label segmentation *e*_*k*_. To each element of *e*_*k*_, i.e. each pixel/voxel *x*, is assigned a label *i* (*e*_*k*_(*x*) = *i*) equal to 0 or 1, for background and segmented object, respectively. A decision matrix *E* is formed with all the *e*_*k*_ vectors, *E* = [*e*_1_ *e*_2_ ⋯ *e*_*K*_] with size *N* × *K*, and fed to a label fusion algorithm to obtain an estimate of the true segmentation *T*.

### 2.2. Data

For evaluating the performance of SVS with respect to STAPLE, Vote and SBA, our data consisted of 2D and 3D simulated data as well as real data.

#### 2.2.1. Two-Dimensional (2D) Simulated Data

We created two simulated 2D data sets: one for training SVS and one for testing the label fusion approaches. The SVS version trained with 2D data is hereafter referred to as SVS-2D.

**Figure 1:**
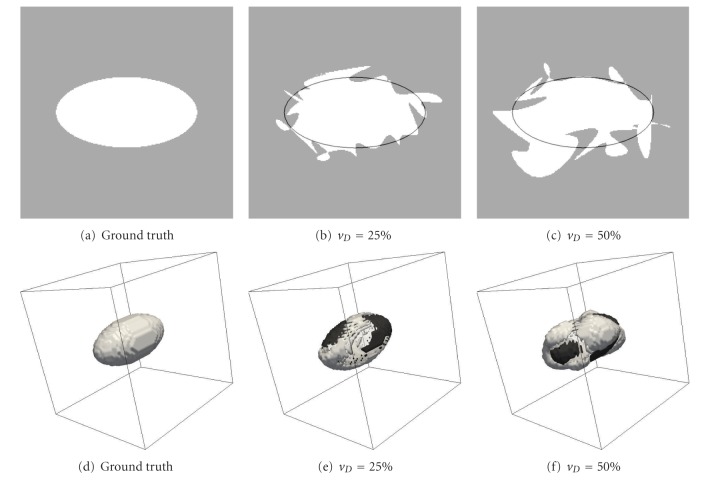
(a, b, and c) 2D and (d, e, and f) 3D simulated images showing the ground truth (a, d), and images with *v*_*D*_ of 25% (b, e) and 50% (c, f). White and black surfaces (e, f) represent, respectively, voxels added to or missing from the ground truth. In 2D, the ground truth was an ellipse geometry of radius 1 AU (arbitrary units) along the *x*-axis and 0.5 AU along the *y*-axis, consisting of eight control points, located at constantly separated angles, between which the ellipse was interpolated with cubic splines. We then mapped this geometry on a grid of 256 × 256 pixels between −1.5 and 1.5 AU along both the *x*- and *y*-axes. In 3D, the ground-truth image was an ellipsoid geometry of radius 1 AU along the *x*-axis and 0.5 AU along both the *y*- and *z*-axes, consisting of 26 control points. See text for construction details. The geometry was mapped in a grid of 64 × 64 × 64 voxels between −1.5 and 1.5 AU along each of the three axes.

The data consisted of multiple binary images created from a ground-truth object, shown in Figure 1(a), which was an ellipse geometry defined by eight control points interpolated with cubic splines.

We generated individual, simulated rater images by moving the control points of the ground-truth ellipse and reinterpolating with cubic splines. We moved the control points in random directions, following a uniform distribution, with random distances from their original coordinates. The random distance followed a normal distribution of zero mean with a standard deviation adjusted so that it could be modified by a normalized deformation factor *f*_*σ*_ (between 0 and 1) to create images with a relative difference area *v*_*D*_ ranging from 0% to 50%, where *v*_*D*_ is given by

(1) $$v_{D}=\frac{v_{k\;|\;T}}{V_{\text{TRUTH}}},$$

where *V*_TRUTH_ corresponds to the area in pixels of the ground-truth ellipse. *v*_*k*∣*T*_ represents the number of pixels in the image that are different between decision *e*_*k*_ of rater *k* and the ground truth *T*:

(2) $$v_{k\;|\;T}=\#\left\{x\;|\;e_{k}\left(x\right)\neq T\left(x\right)\right\}.$$

In other words, *v*_*k*|*T*_ is the total number of false positives and false negatives with respect to *T*. Figures 1(b) and 1(c) show two rater images corresponding to *v*_*D*_ values of 25% and 50%, respectively.

For each of the training and testing sets, we created 625 label fusion tests, each consisting of 10 deformed images, for a total of 6,250 images in the training set and 6,250 different images in the testing set. Each test was created by varying *f*_*σ*_ of the test images according to a given Gaussian distribution. For each test, different mean and standard deviation were used for *f*_*σ*_, ranging both from 0 to 1 with 25 linearly spaced points each, making a total of 625 Gaussian distributions, one for each test. Negative values of *f*_*σ*_ and values higher than 1 were clamped to 0 and 1, respectively. We performed the label fusion of the 10 deformed images in each of the 625 tests of the testing set.

#### 2.2.2. Three-Dimensional (3D) Simulated Data

As for the 2D case, we created two simulated 3D sets: one for training SVS and one for testing the label fusion techniques. The SVS version trained with 3D data is hereafter referred as SVS-3D. An SVS version was also trained with the combination of 2D and 3D training sets. It is referred as SVS-2D&3D.

The 3D data consisted of binary volume images created from a ground-truth ellipsoid. To produce the ground truth, we first created a cubic regular grid volume. This volume was then warped along each axis by dividing each voxel coordinate by its corresponding ground-truth ellipsoid radius, creating a warped grid. By applying this warping transformation, the ellipsoidal space became a spherical space. A ground-truth sphere was created by regularly sampling the angles *θ* and *ϕ* in the spherical-coordinate space (*r*, *θ*, *ϕ*), giving a set of 26 control points (*r*_*c*_, *θ*_*c*_, *ϕ*_*c*_).

To produce the ground-truth image, the control points were projected into a Cartesian space with the following axes: *x* = *θ*, *y* = *ϕ*, and *z* = *r*. We transformed the warped grid into spherical coordinates (*r*_*g*_,  *θ*_*g*_, *ϕ*_*g*_) and performed a cubic interpolation of (*θ*_*g*_, *ϕ*_*g*_) on (*r*_*c*_, *θ*_*c*_, *ϕ*_*c*_) to find r* at each point (*θ*_*g*_, *ϕ*_*g*_). For each grid voxel, if *r*_*g*_ < r*, the voxel was considered inside the sphere and was labeled accordingly. The warped grid (spherical space) was then unwarped into the regular grid (ellipsoidal space) to give the desired ground-truth ellipsoid image shown in Figure 1(d).

While appearing complex, this process in fact simplified the creation of the deformed ellipsoid images. We randomly moved the control points of the ground-truth sphere along *r*, modifying *r*_*c*_, reinterpolated to find r* for the warped grid, performed the labeling by thresholding (i.e. *r*_*g*_ < r*), and unwarped the grid to obtain the deformed ellipsoid.

As for the 2D sets, the random distance followed a normal distribution of zero mean. The standard deviation was adjusted so that it could be modified by *f*_*σ*_ to create deformed ellipsoids with relative difference in volume, *v*_*D*_ (1), ranging between 0% and 50% with respect to the ground truth.

Figures 1(e) and 1(f) show two examples of deformed images with *v*_*D*_ of 25% and 50%, respectively. As for the 2D data, we produced a training set and a testing set, each consisting of 625 label fusion tests. Each test was created as previously described and comprised 10 deformed images. Each of the training and testing sets thus consisted of 6,250 images. We performed the label fusion of the 10 deformed images in each of the 625 tests of the testing set.

#### 2.2.3. Real MRI Data

The real MRI data consisted of intensity images and segmented left and right HC and AG labels of 78 young, neurologically healthy subjects part of the ICBM database [18]. Subjects were scanned in Montréal (Québec, Canada) on a Philips Gyroscan 1.5T scanner (Philips Medical, Best, Netherlands) using a T1-weighted fast gradient echo sequence (sagittal acquisition, TR = 18 ms, TE = 10 ms, 1-mm^3^ voxels, flip angle = 30°). 

The ground truth consisted of left and right HC and AG manual labels, presented in a previous study [19], with a reported intraclass reliability coefficient of 0.900 and 0.925 for interrater and intrarater reliability, respectively.

The labels available for fusion were obtained using a template-based segmentation algorithm [2]. In this approach, each subject's image is compared in turn to a library of other such images; the 10 images with highest match (e.g., highest normalized mutual information) are selected and then nonlinearly aligned with the original subject image. Given that each image in the library has an associated label, inverse warping allows the transfer of label in the original subject's space, where they must be fused to provide a single object. In our dataset, we received 10 labels for each subject, obtained with this technique, for each of the four following regions: left HC, right HC, left AG, and right AG. Label fusions were then performed independently for each region, giving a total of 312 label fusions (78 subjects × 4 regions). We assessed the performance of the fusions using the manual segmentations as “ground truths”.

### 2.3. Label Fusion Strategies

The next sections present the three existing label fusion strategies that we used in this study: STAPLE, Vote, and SBA. We implemented all label fusion methods, including SVS, in MATLAB (MathWorks, Natick, MA, USA).

It is important to note that all approaches were applied to the disputed pixels/voxels only. Pixels/voxels for which all the raters unanimously agreed on their label were not considered; the label was automatically assigned. Working with only disputed pixels/voxels speeded up computation for all methods and significantly improved the results given by STAPLE (see [16]).

#### 2.3.1. STAPLE

STAPLE is an expectation-maximization (EM) algorithm that iteratively estimates (1) the true segmentation from the raters' performance (E-step) and (2) the raters' performance (sensitivity and specificity) from this true segmentation estimate (M-step). We implemented STAPLE following the mathematical description in [20].

#### 2.3.2. Vote

The Vote method consists of summing for each pixel/voxel *x* and label *i*, the occurrences of label *i* among the raters, and assigning the most occurring label to *x*.

#### 2.3.3. SBA

SBA is a voting scheme where each vote is weighted by the signed Euclidean distance computed for each input label. In this study, SBA is the only method that incorporates spatial information in the label fusion process. We implemented this method following the mathematical description in [17].

### 2.4. Label Fusion Strategy Selection: SVS

SVS is a strategy that selects the most appropriate method among STAPLE, Vote, and SBA, based solely on the input labels and their dissimilarity. We point out that SVS is not limited to these three label fusion methods. It could easily be extended to include further methods.

#### 2.4.1. Experimental Observations

**Figure 2:**
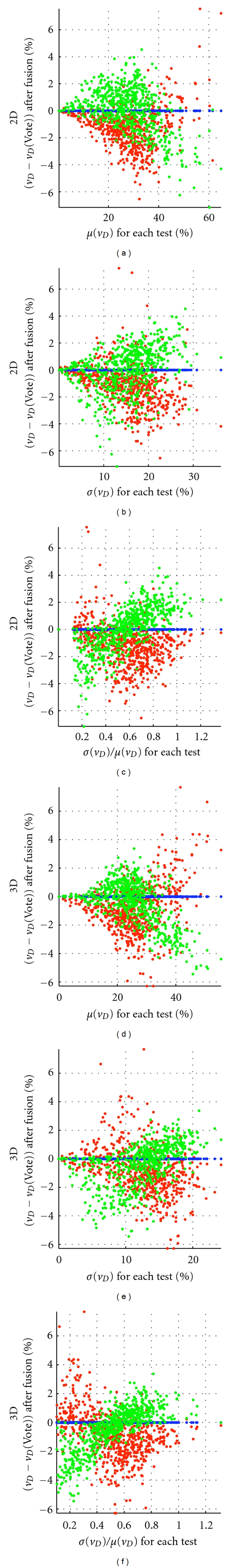
Scatter plots showing *v*_*D*_ centered on the Vote's values, i.e. (*v*_*D*_ − *v*_*D*_(Vote)), obtained after label fusion with STAPLE (red), Vote (blue), and SBA (green) of the 625 tests of the (a, b, and c) 2D and (d, e, and f) 3D training sets, with respect to each test's (a, d) mean (*μ*(*v*_*D*_)), (b, e) standard deviation (*σ*(*v*_*D*_)), and (c, f) coefficient of variation (*σ*(*v*_*D*_)/*μ*(*v*_*D*_)) of *v*_*D*_, calculated over the input labels for each test. The centered *v*_*D*_ corresponds to *v*_*D*_ minus the *v*_*D*_ evaluated for Vote. We note that *σ*(*v*_*D*_)/*μ*(*v*_*D*_) better discriminates the label fusion methods than *σ*(*v*_*D*_).

We developed SVS after observing, during our simulations, that the performance of STAPLE, Vote, and SBA was dependent on the distribution of *v*_*D*_ in the input labels of each label fusion test. This can be observed in the scatter plots of Figure 2 obtained for the 2D (a, b, and c) and 3D training sets (d, e, and f). The scatter plots show *v*_*D*_ centered on the Vote's values, i.e. (*v*_*D*_ − *v*_*D*_(Vote)), after label fusions performed with STAPLE (red), Vote (blue), and SBA (green), as a function of the mean *μ*(*v*_*D*_) (a, d), standard deviation *σ*(*v*_*D*_) (b, e), and coefficient of variation *σ*(*v*_*D*_)/*μ*(*v*_*D*_) (c, f) of *v*_*D*_, calculated over the input labels for each test.

We note that *σ*(*v*_*D*_) and *σ*(*v*_*D*_)/*μ*(*v*_*D*_) give an idea of how differently the raters perform between themselves, while *μ*(*v*_*D*_) measures how bad the raters are overall. These measures thus describe, in a way, the dissimilarity in the raters' input labels.

As can be seen, none of STAPLE, Vote, and SBA can be considered superior to the others. The choice of the best method seems to depend on the distribution of *v*_*D*_. For low values of *σ*(*v*_*D*_)/*μ*(*v*_*D*_), which better discriminates the label fusion methods than *σ*(*v*_*D*_), SBA seems better (i.e. with lower values of *v*_*D*_ after label fusion), while, for higher values, STAPLE would be a better choice. Focusing on the results with respect to *μ*(*v*_*D*_), STAPLE seems better at lower values, and SBA, at higher values. We also observe that in none of the cases does Vote clearly outperform the other methods.

These observations thus suggested that *σ*(*v*_*D*_)/*μ*(*v*_*D*_) and *μ*(*v*_*D*_) could be used to determine the appropriate label fusion method.

#### 2.4.2. Dissimilarity Factors

The measures *σ*(*v*_*D*_)/*μ*(*v*_*D*_) and *μ*(*v*_*D*_) cannot be used in practice since the computation of *v*_*D*_ depends on *v*_*k*∣*T*_ (1) and *V*_TRUTH_, and thus requires to know the ground truth, which is what we try to estimate with label fusion. We thus needed to find estimates for *σ*(*v*_*D*_)/*μ*(*v*_*D*_) and *μ*(*v*_*D*_).

We overcame this problem by using the following scheme. For *v*_*k*∣*T*_, we first computed the frequency of occurrence *f*(*x*, *i*), between 0 and 1, of each label *i* for each pixel/voxel *x* over all raters:

(3) $$f\left(x,i\right)=\frac{\#\left\{k\;|\;e_{k}\left(x\right)=i\right\}}{K}.$$

We then computed, for each rater *k* and each pixel/voxel *x*, the estimated probability that rater *k* misclassifies pixel/voxel *x*, i.e. that the assigned label was a false positive or a false negative:

(4) $$p_{k}\left(x\right)=1-f\left(x,e_{k}\left(x\right)\right).$$

For each estimated rater's probability *p*_*k*_(*x*), we then performed a Bernouilli trial with *B* experiments to compute the probability *P*_*k*_(*x*) that a majority of *B* “virtual” raters misclassified pixel/voxel *x*, according to *p*_*k*_(*x*):

(5) $$P_{k}\left(x\right)=\overset{B}{\underset{i=\left\lceil \left(B+1\right)/2\right\rceil }{\sum }}\left(\begin{array}{c} B\\ i \end{array}\right)p_{k}^{i}\left(x\right){\left(1-p_{k}\left(x\right)\right)}^{B-i}.$$

This last equation corresponds to a cumulative sum of the upper half of the probability mass function of a binomial distribution. In this study, we used *B* = 99 so that *i* ranged from 50 to 99. An odd number for *B* was used to separate the binomial probability mass function equally into a lower and an upper part, the latter corresponding to a clear majority.

From (5), we were able to compute an estimate *v*_*k*_ of *v*_*k*∣*T*_ by summing *P*_*k*_(*x*) over all pixels/voxels:

(6) $$v_{k}=\overset{N}{\underset{x=1}{\sum }}P_{k}\left(x\right).\;$$

To estimate *V*_TRUTH_, we used (3) in a similar Bernouilli trial approach. For each pixel/voxel *x*, we computed a probability that a majority of *B* = 99 “virtual” raters classifies pixel/voxel *x* as being part of the segmented region, i.e. with label 1, according to *f*(*x*, 1):

(7) $$F\left(x\right)=\overset{B}{\underset{i=\left\lceil \left(B+1\right)/2\right\rceil }{\sum }}\left(\begin{array}{c} B\\ i \end{array}\right)f^{i}\left(x,1\right){\left(1-f\left(x,1\right)\right)}^{B-i}.$$

We then summed *F*(*x*) over all pixels/voxels to obtain an estimate *V* of *V*_TRUTH_:

(8) $$V=\overset{N}{\underset{x=1}{\sum }}F\left(x\right).$$

From *v*_*k*_ and *V*, we defined two empirical factors: the *dissimilarity coefficient d*_*c*_, estimating *σ*(*v*_*D*_)/*μ*(*v*_*D*_), and the *dissimilarity ratio d*_*r*_, estimating *μ*(*v*_*D*_). These factors are respectively given by

(9) $$\begin{array}{c} d_{c}=\frac{\sigma \left(v_{k}\right)\;}{\mu \left(v_{k}\right)},\\ d_{r}=\frac{\mu \left(v_{k}\right)}{V}. \end{array}$$

**Figure 3:**
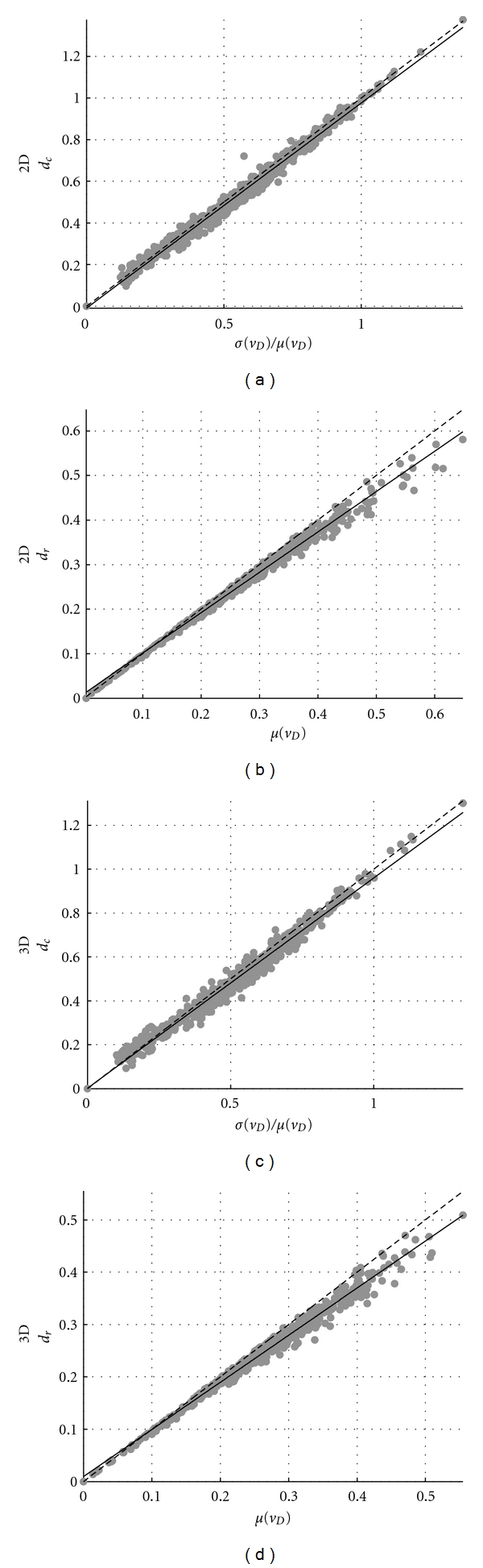
(a, c) *d*_*c*_ versus *σ*(*v*_*D*_)/*μ*(*v*_*D*_) and (b, d) *d*_*r*_ versus *μ*(*v*_*D*_) for all the 625 tests of the (a, b) 2D and (c, d) 3D training sets. A linear fit was performed for both scatter plot types (continuous line) showing the quasi-one-to-one relationship between the theoretical values *σ*(*v*_*D*_)/*μ*(*v*_*D*_), *μ*(*v*_*D*_) and their estimates *d*_*c*_ and *d*_*r*_. The one-to-one relationship is represented by the dashed line.

In Figure 3, we demonstrate the performance of these estimates by showing that *d*_*c*_ (a, c) and *d*_*r*_ (b, d) match, with a quasi-one-to-one relationship, their theoretical values *σ*(*v*_*D*_)/*μ*(*v*_*D*_) and *μ*(*v*_*D*_), respectively, for both the 2D (a, b) and 3D (c, d) training sets.

#### 2.4.3. SVS Training

To perform its selection, SVS finds a score *s*, from the dissimilarity factors *d*_*c*_ and *d*_*r*_, for each of STAPLE, Vote, and SBA, i.e. *s*_STAPLE_(*d*_*c*_, *d*_*r*_), *s*_VOTE_(*d*_*c*_, *d*_*r*_) and *s*_SBA_(*d*_*c*_, *d*_*r*_), and selects the label fusion method that gives the highest score. The following training procedure was used to determine the scoring functions *s*_STAPLE_(*d*_*c*_, *d*_*r*_), *s*_VOTE_(*d*_*c*_, *d*_*r*_), and *s*_SBA_(*d*_*c*_, *d*_*r*_).

1. For each label fusion test *t* of a given training set, we computed *d*_*c*_ and *d*_*r*_, according to the approach presented in the last section.
2. After performing label fusion with STAPLE, Vote, and SBA, we first summed, for each label fusion method *m* and test *t*, the number of pixels/voxels *v*_*m*_ that were different between the label fusion result *T*_*m*_ and the ground truth *T*, i.e. the number of false positives and false negatives:
  (10) $$v_{m}=\#\left\{x\;|\;T_{m}\left(x\right)\neq T\left(x\right)\right\}.$$
  For each test *t*, we assigned a score *s* of 1 to the label fusion method with the lowest *v*_*m*_, corresponding to the best method, 0 to the method with the highest *v*_*m*_, corresponding to the poorest method, and we linearly interpolated the score value for the remaining method.
3. Following the last two steps of the training procedure, we had, for each test *t*, the five following values: *d*_*c*_, *d*_*r*_, *s*_STAPLE_, *s*_VOTE_, and *s*_SBA_. To obtain the continuous scoring functions *s*_STAPLE_(*d*_*c*_, *d*_*r*_), *s*_VOTE_(*d*_*c*_, *d*_*r*_), and *s*_SBA_(*d*_*c*_, *d*_*r*_), we finally fitted, for each method *m*, a surface *s*_*m*_(*d*_*c*_, *d*_*r*_) using locally weighted linear regression (MATLAB Curve Fitting Toolbox, MathWorks, Natick, MA, USA).

This procedure was performed for each of the 2D and 3D training data sets as well as the combination of both sets resulting in three versions of SVS: SVS-2D (trained with 2D data), SVS-3D (trained with 3D data), and SVS-2D&3D (trained with 2D and 3D data). We note that using this scheme, other label fusion methods could be incorporated in SVS, increasing only the number of scoring functions *s*(*d*_*c*_, *d*_*r*_).

**Figure 4:**
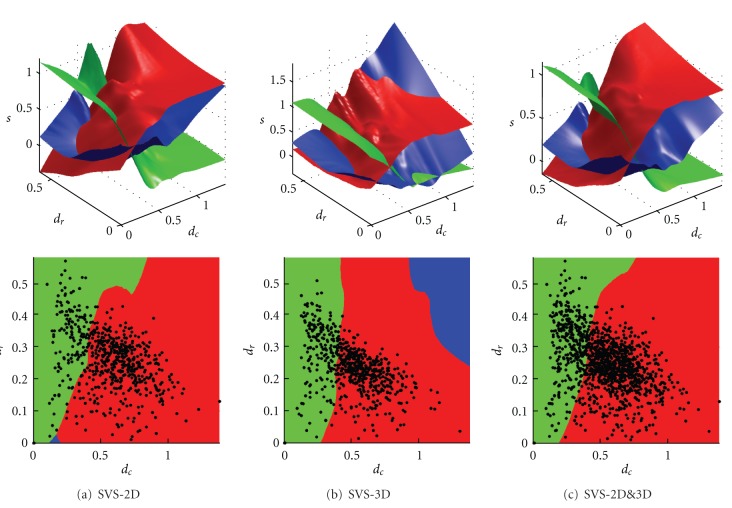
(Top) Scoring surface functions in the space  (*d*_*c*_, *d*_*r*_, *s*) and (bottom) SVS selection regions in the space (*d*_*c*_, *d*_*r*_), where each method among STAPLE (red), Vote (blue), and SBA (green) gives the highest score, for (a) SVS-2D, (b) SVS-3D, and (c) SVS-2D&3D. The bottom images correspond to the top views of the surfaces presented above. The overlaid scatter plot represents the (*d*_*c*_, *d*_*r*_) values of the tests for each SVS version's training set.

Figure 4 presents, for SVS-2D (a), SVS-3D (b), and SVS-2D&3D (c), the scoring surface functions in the space (*d*_*c*_, *d*_*r*_, *s*) as well as the selection regions in the space (*d*_*c*_, *d*_*r*_), where each method gives the highest score. The latter images thus correspond to the top views of the firsts. We observe that the three versions of SVS give very similar delimitations between the methods. Interestingly, with SVS-2D&3D, the border between STAPLE and SBA is almost linear in the region of (*d*_*c*_, *d*_*r*_) covered by the label fusion tests.

#### 2.4.4. SVS Selection

We can now describe the SVS method as follows.

1. Compute the dissimilarity coefficient *d*_*c*_ and the dissimilarity ratio *d*_*r*_ from the raters' input labels, as described in Section 2.4.2.
2. Find the score for each label fusion method using its corresponding scoring surface function.
3. Select the label fusion method corresponding to the highest score.

In case of two or more equal scores, which do not imply identical label fusions, a weighted vote “meta fusion” of the label fusion results, obtained with STAPLE, Vote, and SBA, is performed using the scores as weights. In practice, this situation is uncommon. We point out that, besides the SVS versions presented here, this “meta fusion” approach, i.e. performing a label fusion of STAPLE, Vote, and SBA, has also been tested (results not presented), using each of STAPLE, Vote, and SBA as “meta fusion” method with and without score weights for the two latter methods. However, no “meta fusion” outperformed the versions of SVS presented in this study.

We also point out that *d*_*c*_ and *d*_*r*_ depend on the decision matrix *E* only, i.e. the input labels. Effectively, this ensures that there are no external parameters to the input data that may affect the sensitivity of the technique. Moreover, since *d*_*c*_ and *d*_*r*_ are normalized values, we believe that the technique should not be sensitive to the training data. In fact, we observe in Figure 4 that the different training sets gave similar regions.

### 2.5. Performance Measure

To measure the performance of the label fusion techniques, we computed *v*_*D*_, as well as the Dice similarity coefficient (DSC), an established measure widely reported in the field [1, 2, 9, 11, 12, 15], between each label fusion image and the ground truth. DSC is given by

(11) $$\text{DSC}=\frac{2\left|\text{A}\cap \text{B}\right|}{\left|\text{A}\right|+\left|\text{B}\right|},$$

where |*Z*| is the area or volume of the segmented region *Z*.

**Figure 5:**
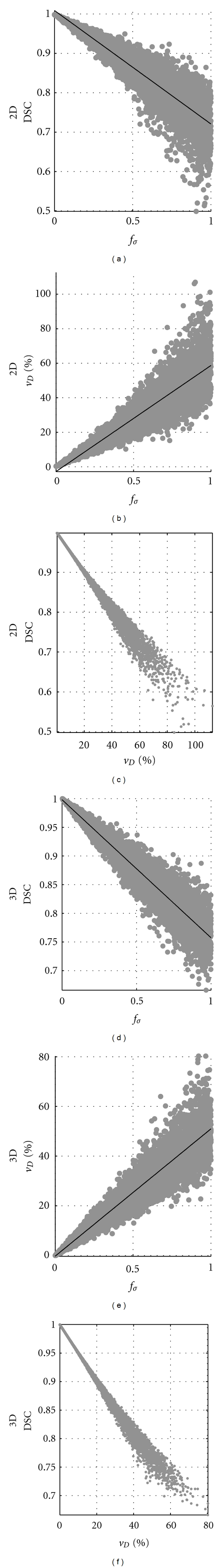
(a, d) DSC and (b, e) *v*_*D*_ for all the 6250 rater images of the (a, b) 2D and (d, e) 3D testing sets as a function of *f*_*σ*_. A linear fit was performed for both scatter plot types (black) showing the quasilinear relationship between *f*_*σ*_ and both DSC and *v*_*D*_. (c, f) DSC plotted as a function of *v*_*D*_. We see that the scatter plots also follow a quasilinear trend. This graph demonstrates that, compared to a point with given *v*_*D*_ and DSC, a neighbor point with a higher (worse) *v*_*D*_ can still give a higher (better) or similar DSC, especially for high *v*_*D*_, questioning the validity of DSC as a performance measure for label fusion.

To further characterize our testing sets and insure the deformation factor *f*_*σ*_ reflected its initial intent, we computed the DSC between each deformed image and its ground truth. Figures 5(a), 5(b), 5(d), and 5(e) show the relationship between DSC, along with *v*_*D*_, and the deformation factor *f*_*σ*_ for the 2D (a, b) and 3D (d, e) testing sets. Figures 5(c) and 5(f) show the quasilinear relationship between DSC and *v*_*D*_.

## 3. Results

### 3.1. 2D Simulated Data

**Figure 6:**
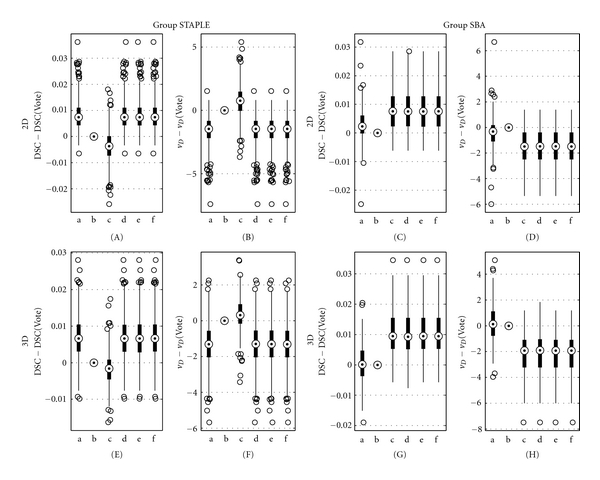
Boxplots of (A, C, E, and G) DSC and (B, D, F, and H) *v*_*D*_, centered on Vote values, for each method for the (A)–(D) 2D and (E)–(H) 3D testing sets. (a) STAPLE, (b) Vote, (c) SBA, (d) SVS-2D, (e) SVS-3D, and (f) SVS-2D&3D. The centered DSC  (*v*_*D*_) corresponds to DSC  (*v*_*D*_) minus DSC  (*v*_*D*_) evaluated for Vote.

**Figure 7:**
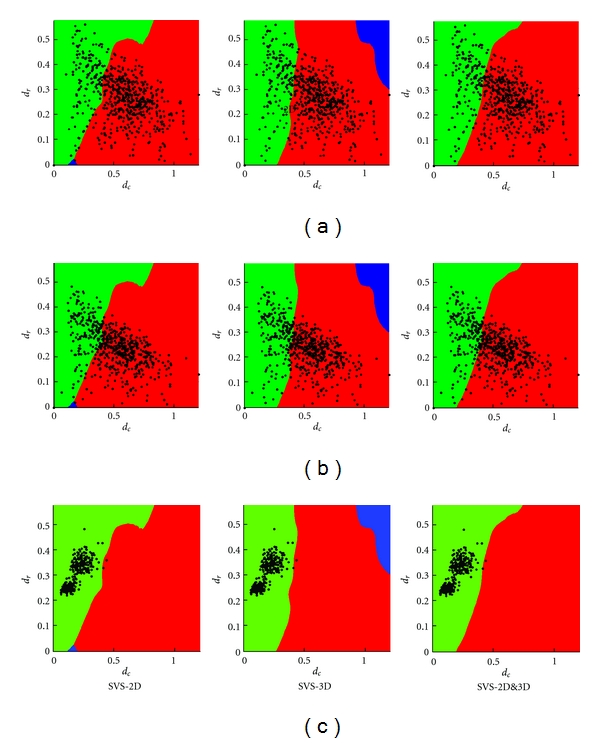
Scatter plots of the (*d*_*c*_, *d*_*r*_) pairs for all the tests of the (a) 2D and (b) 3D testing sets, and (c) real data set, overlaid on the SVS selection regions described in Figure 4 for (left) SVS-2D, (middle) SVS-3D, and (right) SVS-2D&3D. We note that the three versions of SVS selected SBA as the most appropriate method for nearly all tests of the real data set (c).

The three existing techniques (STAPLE, Vote, and SBA) as well as the three versions of SVS (SVS-2D, SVS-3D, and SVS-2D&3D) were used to perform the label fusion of the 10 images of each of the 625 tests of the 2D testing set. Figures 6(A)–6(D) show boxplots of DSC (A, C) and *v*_*D*_ (B, D), centered on the Vote values, obtained with the six fusion methods. To see the improvement brought by SVS (methods d, e, and f), the boxplots have been separated in two groups, *Group STAPLE* and *Group SBA*, determined by the selection performed by SVS-2D&3D on the testing set (see Figure 7(a), right). The data in *Group STAPLE* and *Group SBA* are the tests for which SVS-2D&3D selected STAPLE and SBA, respectively. We see that the SVS boxplots, matching the selected method's, give in both groups higher DSC and lower *v*_*D*_, while each of STAPLE (method a) and SBA (method c) is outperformed in its counterpart group. Regarding Vote (method b), it gives better performance than SBA in *Group STAPLE* but seems to be the worse method in *Group SBA*. We also see that the three versions of SVS are similar despite the different training sets. Figure 7(a) presents the distribution of the (*d*_*c*_, *d*_*r*_) pairs for all the tests of the testing set among the regions of SVS-2D, SVS-3D, and SVS-2D&3D.

### 3.2. 3D Simulated Data

The experiment described in the last section was also performed on the 3D testing set. DSC and *v*_*D*_ boxplots are presented in Figures 6(E, G) and 6(F, H), respectively. The results are very similar to the 2D testing set's; the three versions of SVS give in both groups higher DSC and lower *v*_*D*_.  Figure 7(b) shows the distribution of the (*d*_*c*_, *d*_*r*_) pairs among the selection regions.

### 3.3. Real Data

**Figure 8:**
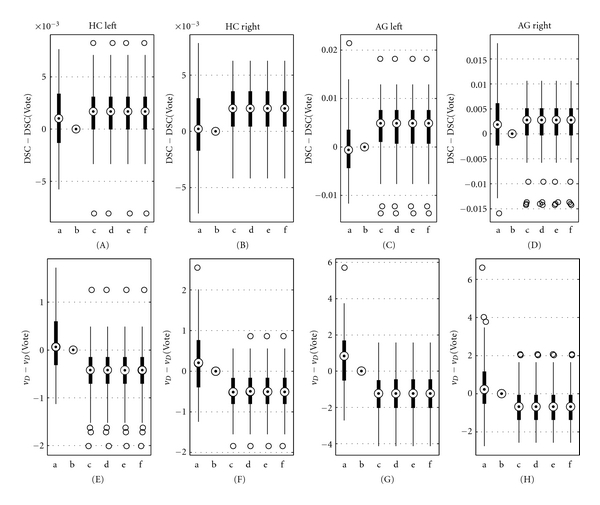
Boxplots of (A)–(D) DSC and (E)–(H) *v*_*D*_, centered on Vote values, for (A, E) HC left, (B, F) HC right, (C, G) AG left, and (D, H) AG right. (a) STAPLE, (b) Vote, (c) SBA, (d) SVS-2D, (e) SVS-3D, and (f) SVS-2D&3D. The centered DSC  (*v*_*D*_) corresponds to DSC  (*v*_*D*_) minus DSC  (*v*_*D*_) evaluated for Vote.

**Figure 9:**
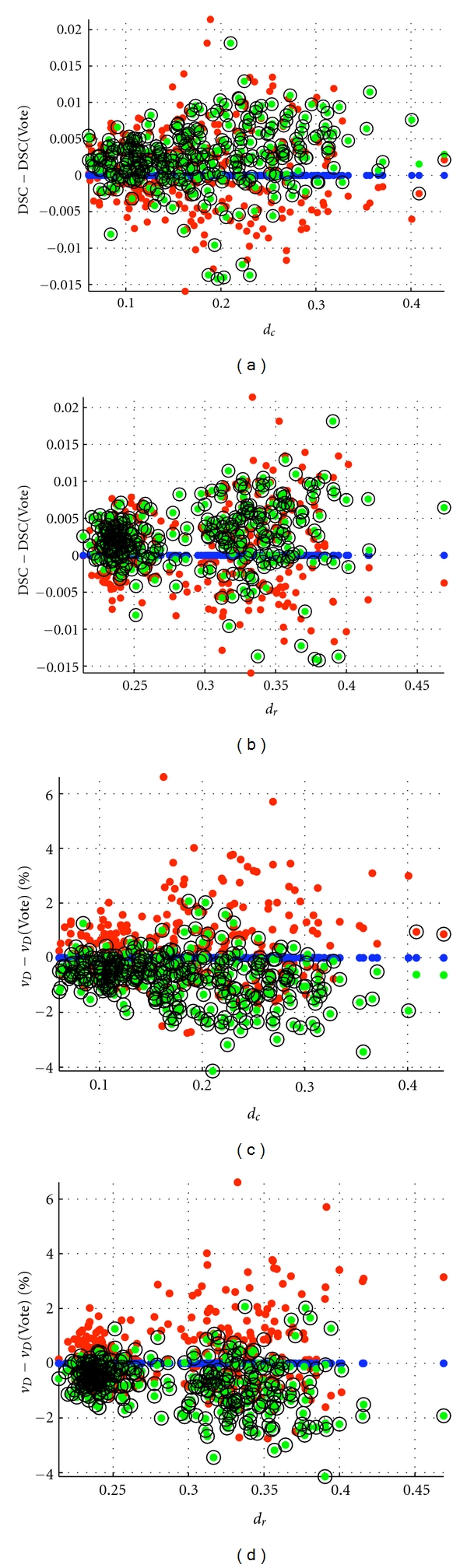
Scatter plots of (a, b) DSC and (c, d) *v*_*D*_, centered on Vote values, as functions of (a, c) *d*_*c*_ and (b, d) *d*_*r*_ for the 312 label fusion tests of the real data set using SVS-2D&3D. The results were nearly identical for SVS-2D and SVS-3D. The centered DSC  (*v*_*D*_) corresponds to DSC  (*v*_*D*_) minus DSC  (*v*_*D*_) evaluated for Vote. DSC and *v*_*D*_ are represented as dots for STAPLE (red), Vote (blue), and SBA (green), and as black circles for SVS-2D&3D.

Figure 8 presents the DSC (A–D) and *v*_*D*_ (E–H) boxplots, respectively, obtained for each of HC and AG, left and right. Since the three versions of SVS (methods d, e, and f) selected SBA for nearly all label fusions, as shown in Figure 7(c), the boxplots are almost identical to SBA's. We see that SBA/SVS overall gives the highest DSC and the lowest *v*_*D*_. This is also shown in Figure 9, which presents scatter plots of DSC (a, b) and *v*_*D*_ (c, d), centered on the Vote values, as a function of *d*_*c*_ (a, c) and *d*_*r*_ (b, d) for all the 312 label fusion cases. SBA/SVS is overall superior to STAPLE and Vote, with DSC and *v*_*D*_ respectively above and below STAPLE and Vote means for the majority of the label fusion cases.

## 4. Discussion

### 4.1. Findings

We showed on a large set of different simulated data that the label fusion method giving the label closest to the ground truth was not the same depending on the dissimilarity among the raters.

Regarding robustness, we showed that SVS outperformed any single method among STAPLE, Vote, and SBA, regardless of the training set. Applying SVS-2D (trained with 2D data) and SVS-3D (trained with 3D data) on 3D and 2D data, respectively, we still obtained better performance than STAPLE, Vote, and SBA. Effectively, the three versions of SVS showed similar results, explained by similar selection regions (Figure 4). This suggests that SVS is independent of the type of training set, 2D or 3D, and that the delimitations of the selecting regions with SVS-2D&3D could represent what we should really expect since there are more training tests.

We also demonstrated that with real data, Vote was not necessarily the method of choice; in our study, SBA was better than Vote and STAPLE. To our knowledge, SBA has not been widely used in the literature, and it might have been underestimated.

### 4.2. Limitations

The first and obvious limitation of the SVS technique is that it is upper-bound limited to the best technique (either STAPLE, Vote, or SBA) in each case.

Secondly, we used DSC and *v*_*D*_ in this study as the criteria for assessing the label fusion methods, the first being commonly used in the literature. However, we think that *v*_*D*_ gives a better indication of the difference between a rater image and the ground truth. This is demonstrated in Figure 8 for HC left, HC right, and AG right. For these regions, while STAPLE's DSC medians are higher (better) than Vote's, *v*_*D*_ medians are higher (worse), meaning that there are more false positives and/or negatives. Also, in Figures 5(c) and 5(f), we show that compared to a point with given *v*_*D*_ and DSC, a neighbor point with a higher *v*_*D*_ (more false positives and/or negatives) can still give a higher (better) or similar DSC, especially for high *v*_*D*_. This difference between DSC and *v*_*D*_ might be explained by the fact that DSC normalizes by the mean area/volume of the label fusion and ground truth, while *v*_*D*_ normalizes by the area/volume of the ground truth only. Therefore, the denominator in *v*_*D*_ remains constant, while the denominator in DSC varies between label fusions. The comparison is thus not performed on the same basis. Although we could argue on which measure is the most appropriate, this questions the validity of DSC as a performance measure for label fusion if the ground truth is available. We thus keep in mind for future work that DSC is not necessarily the best criterion in this case and that *v*_*D*_ should be used instead.

Thirdly, we did not assess the influence of the number and the selection of input labels on the performance of the label fusion strategies. While these two aspects are important, as reported in some studies [2, 12], our objectives were primarily to characterize three existing label fusion strategies and to propose a selection method based on our observations. We will confront these aspects in future work.

## 5. Conclusion

We proposed a method that automatically selects the most appropriate label fusion method based on the dissimilarity of input labels. Overall, the SVS technique performed better with simulated data compared to either individual technique among STAPLE, Vote, and SBA. For real data, SVS selected SBA for almost all cases, which was overall superior to STAPLE and Vote.

## Abbreviations

AD: Alzheimer's disease
AG: Amygdala
AU: Arbitrary units
DSC: Dice similarity coefficient
HC: Hippocampus
MRI: Magnetic resonance imaging
STAPLE: Simultaneous Truth and Performance Level Estimation
SBA: Shape-Based Averaging
SVS: STAPLE-Vote-SBA
2D: Two dimensional
3D: Three dimensional.
